# Supplementary material for: Fluorescent non-canonical amino acid as a site-specific conformational probe of prion formation
Source: bioRxiv. 2026 Apr 24:2026.04.21.719968. Preprint. [Version 1] doi: 10.64898/2026.04.21.719968 (PMC13131685; doi:10.64898/2026.04.21.719968)
Supplement: Supplementary file 1 [file media-1.pdf]

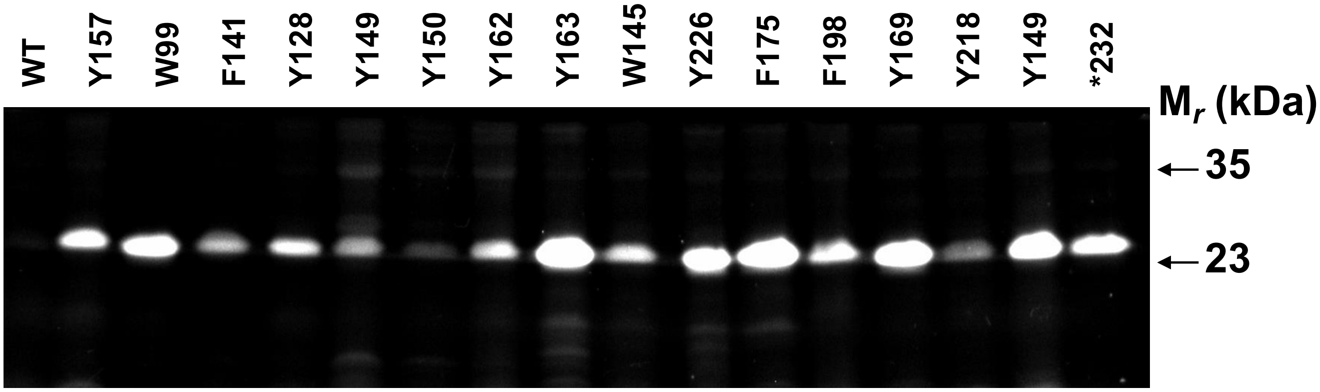

Supplemental Figure 1: Expression of various 7-HCAA mutant PrP molecules in E. coli.
*Fluorescence-imaged SDS polyacrylamide gel of crude lysates prepared from bacteria expressing mutant PrP molecules with 7-HCAA substituted for various residues, as indicated. WT = control bacteria expressing wild-type PrP without 7-HCAA substitution. *232 = amber codon was inserted immediately after the final residue S232 to produce full-length PrP with 7-HCAA added to the C-terminus.*

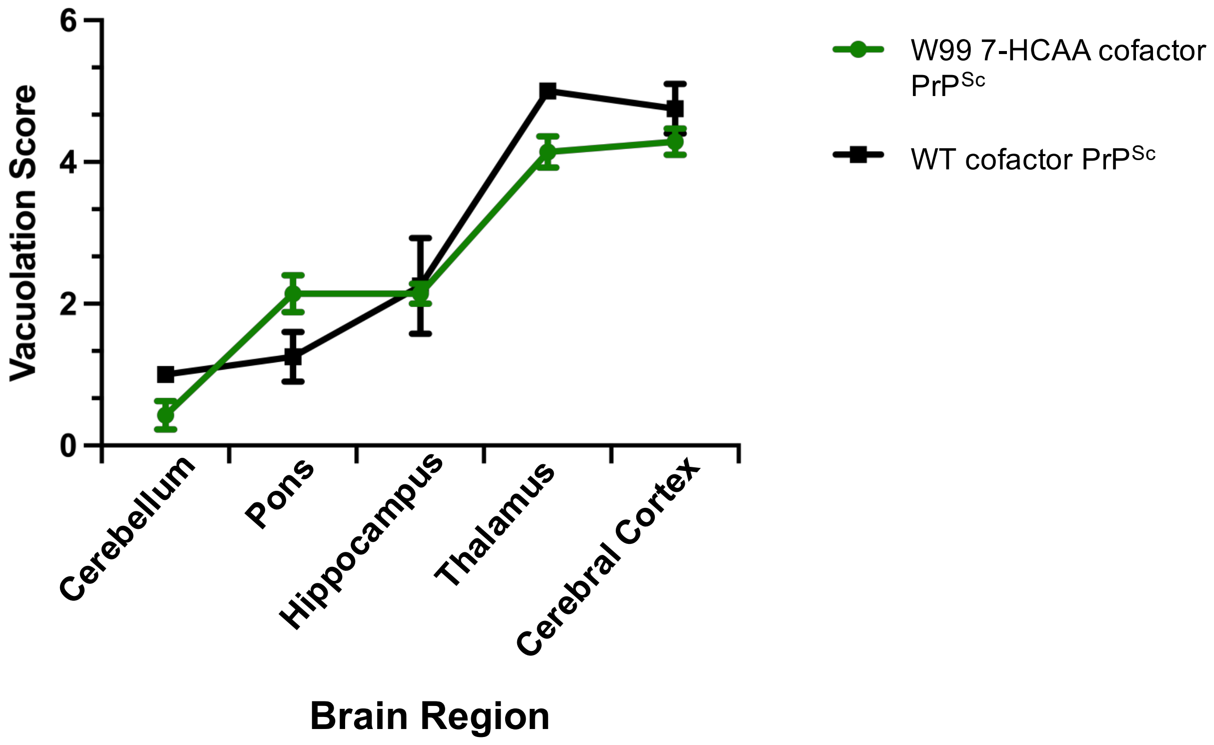

Supplemental Figure 2: Regional neuropathology of kiBV M109 PrP mice inoculated with W99 7-HCAA cofactor PrP^{Sc} and WT cofactor PrP^{Sc}*. Profiles of vacuolation scores of animals inoculated with either W99 7-HCAA cofactor PrP^{Sc} (green circles) or WT cofactor PrP^{Sc} (black squares). Mean values ± SEM are shown.*
